# Microwave Sensors Based on Resonant Elements

**DOI:** 10.3390/s20123375

**Published:** 2020-06-15

**Authors:** Ferran Martín, Paris Vélez, Marta Gil

**Affiliations:** 1 CIMITEC, Departament d’Enginyeria Electrònica, Universitat Autònoma de Barcelona, 08193 Bellaterra, Spain; paris.velez@uab.cat; 2 Departamento de Ingeniería Audiovisual y Comunicaciones, Universidad Politécnica de Madrid, 28031 Madrid, Spain; marta.gil.barba@upm.es

**Keywords:** microwave sensors, planar sensors, resonant sensors, internet of things (IoT), humidity sensors, permittivity sensors, microfluidics, substrate integrated waveguides (SIW), electrically small resonators, differential-mode sensors, frequency-variation sensors, phase-variation sensors, coupling-modulation sensors, frequency-splitting sensors

## Abstract

This paper highlights interest in the implementation of microwave sensors based on resonant elements, the subject of a special issue in the journal. A classification of these sensors on the basis of the operating principle is presented, and the advantages and limitations of the different sensor types are pointed out. Finally, the paper summarizes the different contributions to the special issue.

## 1. Introduction

Within the framework of the Internet of Things (IoT) paradigm, there is an increasing demand for sensing using microwave signals. Their low cost, high sensitivity, robustness against harsh environments, and their potential for non-invasive and wireless sensing are advantageous aspects of microwave sensors. Among them, planar resonant sensors have attracted the attention of many researchers in recent years. Since resonant elements are sensitive to the properties of their surrounding medium, microwave sensors based on such elements have been applied to many different scenarios, including material characterization [1,2,3], bio-sensing [4], ambient monitoring [5], defect detection [6], motion control [7,8], chemical analysis [9], microfluidics [10,11], etc. Moreover, sensor implementation in planar technology is interesting for various reasons, including the possibility of developing low-profile and low-cost sensors, conformal sensors (e.g., in flexible substrates) [12], recyclable sensors (e.g., based on organic or compostable substrates), wearable sensors [13], integrated sensors [14], submersible sensors [15], or sensors compatible with other technologies (e.g., microfluidics [10,11], substrate-integrated waveguide-based sensors [16], lab-on-a-chip sensors [17], etc.).

Planar microwave resonant sensors can be classified according to various criteria, including frequency of operation, field of application, or operation principle, among others. However, probably the most convenient scheme for sensor categorization is their working principle, since it eases sensor comparison [18]. That is, comparing sensor performance in terms of the relevant sensor parameters, such as sensitivity, resolution, dynamic range, linearity, etc., is difficult if the devices work under different operational principles. Nevertheless, it should be mentioned that there are sensors in which several working principles are exploited simultaneously. According to their principle of operation, microwave resonant sensors can be divided into: frequency-variation sensors [1,2,3,19,20,21,22,23,24,25,26,27,28,29,30,31], phase-variation sensors [6,32,33,34,35], frequency-splitting sensors [36,37,38,39,40,41,42,43,44,45], coupling-modulation sensors [46,47,48,49,50,51,52,53,54,55,56] and differential-mode sensors [6,33,35,57,58,59,60,61,62,63,64,65,66,67].

In frequency-variation sensors, the variable of interest (measurand) modifies the resonance frequency and magnitude (either a peak or a notch, depending on the specific implementation) of the sensing element, based on a resonator. Several specific implementations can be considered, including a transmission line loaded with a single or with multiple resonators, or a resonator-loaded antenna, among others. Such sensors are simple and easy to design, and they are very common. Frequency-variation sensors are of special interest for material characterization, as the resonance frequency of the sensing resonators is perturbed by the presence of a material in contact or in close proximity to it [1,2,3,22]. However, frequency-variation sensors need wideband interrogation signals for measuring purposes, especially in scenarios with significant input dynamic ranges, and this penalizes the cost associated with the electronics necessary for the generation of such signals. Moreover, frequency-variation sensors are subjected to cross-sensitivities, e.g., caused by changes in environmental factors.

Phase-variation sensors typically operate at a single frequency, and thereby constitute a good solution to reduce the cost of the electronics (a single-tone, or harmonic, signal suffices for measuring purposes). However, measuring the phase of a signal is not always simple, at least as compared to the magnitude. For this reason, phase-variation sensors based on phase-to-magnitude converters have been proposed [64]. Phase-variation sensors based on ordinary meandered lines with very high sensitivities have been demonstrated [33] at the expense of large sensing areas. However, thanks to the controllability of the dispersion diagram in artificial lines, highly sensitive and small-sized phase-variation sensors based on composite right/left handed lines [35], slow-wave transmission lines [34] and electroinductive-wave (EIW) transmission lines [6] have been reported. The most canonical applications of these sensors are dielectric constant measurements and material characterization. However, phase-variation sensors devoted to the measurement of spatial variables, specifically angular displacements and velocities, have also been reported [68,69].

To alleviate the cross-sensitivities caused by the effects of ambient factors (e.g., temperature, humidity, etc.), sensors based on symmetry truncation and differential-mode sensors constitute a good solution. There are two main groups of sensors based on symmetry properties, i.e., coupling-modulation sensors and frequency-splitting sensors. In coupling-modulation sensors, a transmission line is symmetrically loaded with a symmetric resonator. Under perfect symmetry, the resonator-loaded line is transparent to signal propagation, provided the symmetry planes of the line and resonator are of a different electromagnetic sort (one a magnetic wall and the other one an electric wall) [46,70]. However, when symmetry is truncated, e.g., by means of a relative displacement between the line and the resonator, or by means of an asymmetric dielectric loading of the resonant element, then a notched response arises, and the magnitude of the notch can be used for sensing, since it is intimately related to the level of asymmetry caused by the input variable (typically, but not exclusively, a displacement). By contrast, in a frequency splitting sensor, a line is symmetrically loaded with a pair of identical resonators. Under perfect symmetry, a single notch in the frequency response arises. However, when symmetry is disrupted as consequence of a perturbation caused by the input variable, the original notch is split into two notches, whose separation is related to the level of asymmetry. Typically, these sensors have been applied to dielectric characterization [42,43]. As compared to the previous coupling modulation sensors, frequency-splitting sensors do also require wideband signals in order to carry out measurements. In both sensor types, coupling-modulation and frequency splitting, symmetry is preserved under changes in environmental factors, as far as such changes, if present, occur at scales much higher than the typical dimensions of the sensors. Thus, such sensors are robust against cross-sensitivities caused by ambient factors. 

Differential-mode sensors are based on two independent sensing elements and are also robust against changes in environmental factors, since such changes are seen as common-mode stimulus by such sensors. There are many types of differential-mode sensors based on resonant elements. In some implementations a pair of lines is loaded with a pair of identical resonators, and the output variable is the cross-mode transmission coefficient, indicative of the differences between the transmission coefficients in both sensing lines [58,59,60,61]. Typically, these sensors are based on some of the principles discussed before, e.g., phase or frequency variation. Several examples of differential-mode sensors devoted to liquid characterization, and implemented by adding fluidic channels on the sensitive regions, have been reported in the literature [58,59,60,61,62]. It should also be mentioned that differential-mode sensors based on reflection, rather than transmission, have been also proposed [65,66]. In these latter sensors, the number of ports is reduced to two, and, typically, these sensors are smaller, and especially suited in certain applications, e.g., submersible sensors. 

## 2. Brief Summary of the Special Issue Papers

The special issue includes six papers, based on different configurations, and devoted to different applications. Such papers are representative of the latest developments in the field.

In [71], a frequency-splitting sensor for dielectric characterization based on a transmission line loaded with a pair of identical magnetic LC resonators (inductance-capacitance resonator) is reported. The sensitivity of the sensor is enhanced by removing the mutual coupling between the two halves of the magnetic-LC resonator using a metallic wall. A mathematical sensing model is developed for measuring the relative permittivity of unknown samples based on the frequency-splitting principle. The measurements and comparisons with state-of-art sensors in the literature demonstrate the high performance of the designed sensor in determining the relative permittivity of unknown solid dielectric materials.

In paper [72], a frequency-variation reflective-mode sensor based on a planar patch antenna acting as resonant element is proposed. The sensor is aimed at water content monitoring in sediments by immersing the antenna. The sensor operates in reflection mode by monitoring the variation of the resonant frequency as a function of the sediment density through the reflection coefficient. The developed sensor can be envisaged as a promising innovative device for the monitoring of sediments in geological sciences.

Paper [73] proposes a method for the measurement of the complex dielectric constant of solid slabs that combines three CSRRs (complementary split-ring resonators) with different orientations with a substrate integrated waveguide (SIW). Such combination results in a sensor with high sensitivity and wide input dynamic range. The frequency selectivity of the sensor is improved by the high-quality factor of the SIW. The convenient orientations of the CSRRs for sensitivity improvement are discussed in the paper.

A permittivity sensor based on a CSRR is presented in paper [74]. The intended application is the determination of soil water content. The specific implementation is a circular microstrip patch antenna and the working principle of operation is based on the shifting of two of the antenna resonant frequencies caused by changing the relative permittivity of the material under test (MUT), i.e., frequency variation. The effectiveness of the sensor is demonstrated by characterizing typical dielectric materials. Sensor validation is carried out by determining the percentage of water concentration in quartz sand and red clay samples.

In [75], an ultra-compact humidity sensor based on a double-folded SIW re-entrant cavity is proposed and analyzed. By folding a circular re-entrant cavity twice along its two orthogonally symmetric planes, the authors are able to achieve a remarkable size reduction (up to 85.9%) in comparison with a conventional TM_010_-mode circular SIW cavity. The operating principle of the humidity sensor is based on the resonant method, and it utilizes the resonant properties of the sensor as signatures to detect the humidity condition of the ambient environment. To this end, a mathematical model quantitatively relating the resonant frequency of the sensor and the relative humidity level is established according to the cavity perturbation theory. The sensor demonstrates very good sensitivity and benefits from a high Q-factor and ease of fabrication and integration. These advantages make it an excellent candidate for humidity-sensing applications in various fields such as the agricultural, pharmaceutical, and food industries, as pointed out by the authors.

Finally, paper [60] reports a differential-mode sensor based on a pair of lines loaded with dumbbell-shaped defect ground structure resonators. The sensor includes a pair of microfluidic channels and is devoted to liquid characterization. In particular, two applications are reported in the paper, i.e., the measurement of electrolyte concentration in deionized water, and the characterization of solute content in solutions of isopropanol in deionized water. A detailed sensitivity analysis useful for sensor design, and based on the equivalent circuit model of the sensor, is carried out in the paper. Such analysis links the input variables with the cross-mode transmission coefficient, the output variable.
